# Supplementary material for: In vitro Edwardsiella piscicida CK108 Transcriptome Profiles with Subinhibitory Concentrations of Phenol and Formalin Reveal New Insights into Bacterial Pathogenesis Mechanisms
Source: Microorganisms. 2020 Jul 17;8(7):1068. doi: 10.3390/microorganisms8071068 (PMC7409036; doi:10.3390/microorganisms8071068)

Supplementary Fig 1. Determination of the distance between inter- and intragroup. (A) Hierarchical clustering of samples by the Euclidean distances. The distance reflects on the histogram color as indicated as color key. (B) PCA plot.

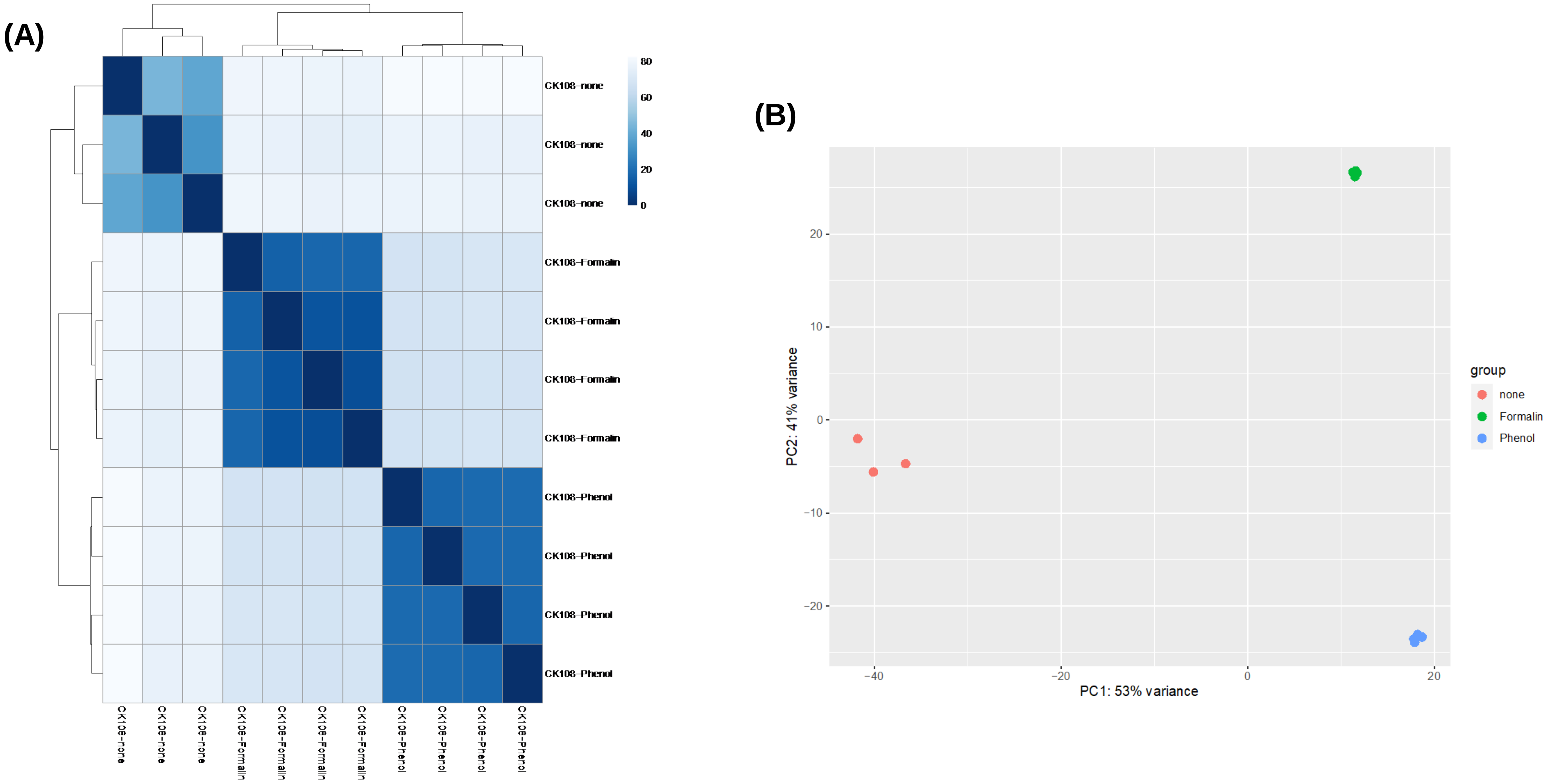

Supplement: Supplementary file 1 [file microorganisms-08-01068-s001.zip › Suppl_Figure.pdf]
